# Unraveling Chagas disease transmission through the oral route: Gateways to *Trypanosoma cruzi* infection and target tissues

**DOI:** 10.1371/journal.pntd.0005507

**Published:** 2017-04-05

**Authors:** Danielle Silva-dos-Santos, Juliana Barreto-de-Albuquerque, Bárbara Guerra, Otacilio C. Moreira, Luiz Ricardo Berbert, Mariana Tavares Ramos, Barbara Angelica S. Mascarenhas, Constança Britto, Alexandre Morrot, Déa M. Serra Villa-Verde, Luciana Ribeiro Garzoni, Wilson Savino, Vinícius Cotta-de-Almeida, Juliana de Meis

**Affiliations:** 1 Laboratory on Thymus Research, Oswaldo Cruz Institute, Oswaldo Cruz Foundation, Rio de Janeiro, Brazil; 2 National Center of Structural Biology and Bio-imaging—CENABIO, Federal University of Rio de Janeiro, Rio de Janeiro, Brazil; 3 Laboratory on Molecular Biology and Endemic Diseases, Oswaldo Cruz Institute, Oswaldo Cruz Foundation, Rio de Janeiro, Brazil; 4 Department of Immunology, Microbiology Institute, Federal University of Rio de Janeiro, Rio de Janeiro, Brazil; 5 Laboratory for Innovations in Therapies, Education and Bioproducts, Oswaldo Cruz Institute, Oswaldo Cruz Foundation, Rio de Janeiro, Brazil; Yeshiva University Albert Einstein College of Medicine, UNITED STATES

## Abstract

Oral transmission of *Trypanosoma cruzi*, the causative agent of Chagas disease, is the most important route of infection in Brazilian Amazon and Venezuela. Other South American countries have also reported outbreaks associated with food consumption. A recent study showed the importance of parasite contact with oral cavity to induce a highly severe acute disease in mice. However, it remains uncertain the primary site of parasite entry and multiplication due to an oral infection. Here, we evaluated the presence of *T*. *cruzi* Dm28c luciferase (Dm28c-luc) parasites in orally infected mice, by bioluminescence and quantitative real-time PCR. *In vivo* bioluminescent images indicated the nasomaxillary region as the site of parasite invasion in the host, becoming consistently infected throughout the acute phase. At later moments, 7 and 21 days post-infection (dpi), luminescent signal is denser in the thorax, abdomen and genital region, because of parasite dissemination in different tissues. *Ex vivo* analysis demonstrated that the nasomaxillary region, heart, mandibular lymph nodes, liver, spleen, brain, epididymal fat associated to male sex organs, salivary glands, cheek muscle, mesenteric fat and lymph nodes, stomach, esophagus, small and large intestine are target tissues at latter moments of infection. In the same line, amastigote nests of Dm28c GFP *T*. *cruzi* were detected in the nasal cavity of 6 dpi mice. Parasite quantification by real-time qPCR at 7 and 21 dpi showed predominant *T*. *cruzi* detection and expansion in mouse nasal cavity. Moreover, *T*. *cruzi* DNA was also observed in the mandibular lymph nodes, pituitary gland, heart, liver, small intestine and spleen at 7 dpi, and further, disseminated to other tissues, such as the brain, stomach, esophagus and large intestine at 21 dpi. Our results clearly demonstrated that oral cavity and adjacent compartments is the main target region in oral *T*. *cruzi* infection leading to parasite multiplication at the nasal cavity.

## Author summary

Oral transmission of *Trypanosoma cruzi* associated with food/beverage consumption is presently an important route of infection in Brazil and Venezuela. Colombia, Bolivia, Argentina and Ecuador have also reported to have acute cases of Chagas disease transmission through the oral route. Significant studies about this form of *T*. *cruzi* infection are largely lacking. In addition to the classic cardiac involvement, orally-infected patient progress to a highly symptomatic disease and increased mortality rate (8–35%), surpassing the calculated mortality produced by the disease resulting from the biting of infected insect vectors (5–10%). Here, we explored by in vivo bioluminescent images, qPCR and fluorescence microscopy the primary site of parasite entry and multiplication in oral infection (**OI)**. Our results clearly demonstrated that the oral cavity is the main *T*. *cruzi* target region in **OI**, leading to parasite multiplication at the nasal cavity and parasite dissemination to the brain and peripheral tissues. Interestingly, facial edema, paraesthesia of the tongue, gingivitis and dry cough were already described in affected patients. These findings might be associated to our present data, which describe for the first time the nasomaxillary region as the main target tissue following oral *T*. *cruzi* infection.

## Introduction

Human Chagas disease (American trypanosomiasis) is a neglected tropical illness caused by the protozoan *Trypanosoma cruzi*. Infection affects 6–8 million people worldwide and is considered a global health problem. Chagas disease is endemic in Mexico, Central America and South America and is also spreading in non-endemic countries through migration of infected people [1]. It can be transmitted by excreta deposition after biting of blood sucking *Triatominae* bugs, blood transfusion; organ transplantation; laboratory accident; congenitally and orally [2, 3].

Outbreaks of oral transmission of Chagas disease were described in Brazil, Venezuela, Colombia, French Guyana, Bolivia, Argentina and Ecuador [4–9]. All of these outbreaks were associated with contaminated food/beverage consumption as wild animal meat, vegetables, sugar cane extract, açaí pulp, guava juice, bacaba, babaçu and vino de palma [10–12]. From 1968 to 2000, 50% of acute cases in Amazon region were attributed to oral transmission [8] and these numbers reached 70% between 2000–2010 [6]. Venezuela has also reported the biggest outbreak described so far, with two distinct occurrences affecting respectively 103 and 88 people. These outbreaks involved adults and children from urban and rural schools [5, 13]. Mortality rate in orally infected patients is reported as higher (8–35%) when compared to the classical vectorial transmission, through triatomine excreta deposition after biting (<5–10%) [14]. It is well known that both trypomastigotes and metacyclic trypomastigotes are associated with oral Chagas transmission [15–17]. Regarding *T*. *cruzi* genotypes, isolates from DTUs I, II, III, IV and VI have been associated with patients from oral Chagas outbreaks [18–25]. Although relevant, there are few reports about *T*. *cruzi* oral transmission in the literature. Some authors have demonstrated parasite-mucosa interaction, some aspects of immune response as well as disease outcome after intragastric, pharyngeal or buccal parasite challenge. These models of oral *T*. *cruzi* infections present both patent parasitemia and heart parasitism, which indicate systemic infection [26–30]. In addition, *T*. *cruzi* glycoprotein gp82 seems to bind gastric mucin, promoting invasion and replication in epithelial cells from the gastric mucosa [31]. This initial invasion is related to establishment of a progressive gastritis and allows further systemic dissemination of the parasite. Nonetheless, the short replication period at this mucosal site induces specific immunity, as protection was observed after a secondary mucosal challenge, involving the production of IgA and IgG antibodies [27]. In orally infected mice, inflammatory infiltrates are observed in tissues such as pancreas, spleen, liver, bone marrow, heart, duodenum, adrenal glands, brain and skeletal muscle. Moreover, it was suggested that intraepithelial and lamina propria lymphocytes are involved in IFN-γ but not IL-4 production in orally infected hosts [27]. Following disease outbreaks caused by *T*. *cruzi* food contamination, a clear increase in severity of clinical manifestations was observed in patients, as compared with other types of transmission routes [8, 14]. These observations raise important questions concerning the particular features of *T*. *cruzi* entry via the mucosa, including the possible modulation of local immune mechanisms and the impact on regional and systemic immunity [32, 33]. We have recently demonstrated that the site of parasite entrance, through oral infection (**OI**)–directly in the mouth, as observed in natural infection, or gastrointestinal infection (**GI**)–directly to the stomach via gavage differentially affects host immune response and mortality. Thus, comparing to **GI** mice, we observed that **OI** mice presented elevated infection rate and parasitemia, higher TNF serum levels, more severe hepatitis and milder carditis [15]. This difference in immunological response and infection severity between **GI** and **OI** mice raised important questions about the primary site of *T*. *cruzi* infection by the oral route and its impact on disease progression.

Bioluminescent imaging is a promising technique that brings the opportunity to approach the *in vivo* host-pathogen interactions through a highly sensitive and non-invasive way [34]. In addition to allow the follow up of infection progression by keeping the animal alive, this technique also gives the possibility to observe new sites of infection and parasite distribution that are hardly observed by histological techniques [35]. In the past years, some reports developed *in vivo* bioluminescent analysis both in *T*. *cruzi* infected mice and in the invertebrate host [35–37]. In the present work, by employing the bioluminescent technique and real-time qPCR, we followed the dynamics of *T*. *cruzi* Dm28c luciferase (Dm28c-luc) distribution throughout the host using our well-established model of **OI** in mice [15]. The bioluminescence results indicated the nasal cavity as the main primary site of parasite invasion and multiplication in the host. At later moments, luminescent signal progressively increased in the abdomen and genital region, as a result of parasite dissemination. Quantification of parasite load, via *T*. *cruzi* satellite DNA (SatDNA) detection by real-time qPCR at 7 and 21 dpi, corroborated the bioluminescence results, showing predominant *T*. *cruzi* detection in mouse nasal cavity. Parasite amplification was also observed in the mandibular lymph nodes, pituitary gland, heart, liver, small intestine and spleen at 7 dpi, and was disseminated to other tissues, such as the brain, stomach, esophagus and large intestine at 21 dpi. Our results indicate the oral cavity and adjacent tissues as the main target region for oral *T*. *cruzi* infection, leading to parasite multiplication at the nasal cavity.

## Methods

### Mice and *Trypanosoma cruzi* infection

Male BALB/c mice, aged 6–8 weeks, were obtained from the animal facility of Oswaldo Cruz Foundation (Rio de Janeiro, Brazil) and used in all experiments. Animals were handled according to the rules of the Ethics Committee for Animal Research of Oswaldo Cruz Foundation. The total number of mice used in each experimental set is described in S1 Fig flowchart. Mice were infected via the oral cavity (**OI**) with trypomastigotes of a Dm28c (DTU- TcI) genetically modified to express the firefly luciferase (Dm28c-luc), Dm28c-GFP or Tulahuén (DTU- TcVI) strains [35, 38].

Parasites were obtained from infected cultures of a monkey kidney epithelial cell line (Vero cells) from the particular Cell Line Collection of the Laboratory on Thymus Research, Oswaldo Cruz Institute. *T*. *cruzi* were counted using Neubauer's chamber in phosphate buffered saline (PBS). Mice were maintained starving for 4 hours and then infected with 1x10^6^ trypomastigotes in 50 μL of parasite suspension into the mouth. At the infection moment, mice swallowing time was respected to avoid parasite aspiration.

A control experiment was performed with injection of 50 μL of black ink suspension at the oral cavity or intranasally to validate our protocol of oral infection and to exclude the possibility of an intranasal contamination (S2 Fig).

### Ethics statement

This study was performed in strict accordance with the recommendations in the Guide for the Care and Use of Laboratory Animals of the Brazilian National Council of Animal Experimentation and the Federal Law 11.794 (10/2008). The Institutional Ethics Committee for Animal Research of the Oswaldo Cruz Foundation (CEUA-FIOCRUZ, License: LW-23/12) approved all the procedures used in this study.

### Parasitemia

Mouse parasitemia was individually evaluated at different days post-infection (4, 7, 11, 14 and 21 dpi–S1 Fig) by counting trypomastigotes in 5 μL of tail vessels blood. Blood-parasite number was calculated according to the Brenner method.

### *In vivo* and *ex vivo* bioluminescence imaging (BLI)

Photoluminescence signals were measured at different time points post-infection (15 and 60 minutes (min), 7 and 21 dpi–S1 Fig), in anesthetized animal, by ventral and lateral position using the IVIS Lumina image system (Xenogen Corp, CA, EUA). D-luciferin potassium salt (Xenogen) stock solution was prepared in PBS at 15 mg/mL and stored at -80°C.

Analyses of 15 min post-infection imaging were performed with a 5 min pre-incubation of Dm28c-luc with 0.15 mg in PBS (10 μL) of D-luciferin stock solution followed by mouse infection. Photoluminescent images of infected mice were acquired 15 min later.

Images at 60 min post-infection were carried out after intraperitoneal injection of D-luciferin (150 mg/Kg of body weight) followed by an addition of 50 μL of D-luciferin (0.75 mg in PBS) at the oral cavity, just before capturing the images.

At 7 and 21dpi analyses, photoluminescent signals were measured with images starting 15 min after an intraperitoneal injection of D-luciferin solution in potassium salt (150 mg/Kg of body weight).

Mice were placed inside the animal chamber anaesthesia delivery system (Xenogen XGI-8 Gas Anaesthesia system). Isoflurane (1.5%) anaesthesia was applied until the mice became recumbent. These animals were then placed into the image chamber of IVIS Lumina system (Xenogen Corp, CA, USA) and controlled flow of isoflurane, with a nose cone device into the chamber, maintained them anesthetized during the bioluminescence imaging acquisition.

For the analysis of *T*. *cruzi* presence in specific organs, mice were injected with D-luciferin at different times post-infection (S1 Fig), and 10 min later mice were euthanized in order to perform single tissue harvest. Tissues were removed, transferred to a culture dish and images acquired at the IVIS Lumina image system.

Acquisition of bioluminescent images of both mice and tissues was performed by 5 min of exposure and the photons emitted from luciferase-expression *T*. *cruzi* were quantified using the Living Image 3.0 software program.

### *Ex vivo* analysis of parasite nests

Uninfected and six days post infection mice were euthanized, the nasal cavity were isolated and tissue were included in tissue tek (OCT, Sakura, USA). Cryosections (5 μm) of frozen tissues were analyzed using a fluorescent Zeiss microscope (Germany). Images were digitalized using AxioCam HRm and Axio Vision Rel 4.8 software.

### DNA extraction from tissues

DNA extraction was performed from nasal cavity, palate, tongue, esophagus, stomach, small intestine, large intestine, liver, heart, spleen, mandibular lymph nodes, pituitary gland and brain, using the QIAamp DNA Mini kit (Qiagen, CA). Tissues were obtained from dissected infected mice at different time points (60 min, 7 and 21 dpi), individually weighted (maximum 10 mg for spleen and 30 mg for other tissues was used), washed in PBS (except tissues from nasal cavity, mandibular lymph nodes and pituitary gland) and stored at -20°C until DNA extraction. Blood was drawn via cardiovascular perfusion with PBS, immediately after euthanasia. Nasal cavity tissue was obtained after scraping the region. Tissues and organs from non infected mice were used for negative control. The protocol was carried out according to the manufacturer’s instructions and the DNA was eluted with 100 μL of elution buffer (AE). As a qualitative internal reference control, the exogenous internal amplification control (IAC), a pZErO-2 plasmid containing an insert from the A. thaliana aquaporin gene, was used as reported by Duffy (2009). Before DNA extraction, 5 μL (40 pg/mL) of linearized IAC were added to the samples. DNAs were stored at −20 ◦C until use and their purity and concentration were determined using a Nanodrop 2000c spectrophotometer (Thermo Scientific) at 260/280 and 260/320 nm.

### Quantitative duplex real-time PCR (qPCR)

According to the international consensus for quantification of *Trypanosoma cruzi* DNA in Chagas disease patients [39], Quantitative Real Time PCR Multiplex assays using TaqMan probes were performed targeting the satellite region of the nuclear DNA (SatDNA) of *T*. *cruzi* and the exogenous internal amplification control (IAC), as described in Duffy et al, 2009. The qPCR reactions were performed in a final volume of 10 μL containing 1.5 μL of DNA template, 5 μL of 2X TaqMan Universal PCR Master Mix (Applied Biosystems, USA), 750 nM of both cruzi1 (5′ASTCGGCTGATCGTTTTCGA 3′) and cruzi2 (5′AATTCCTCCAAGCAGCGGATA3′) primers and 50 nM cruzi3 probe (5′FAM- CACACACTGGACACCAA-NFQ-MGB 3′) specific for *T*. *cruzi* SatDNA; 100 nM IAC Fw (5′CCGTCATGGAACAGCACGTA3′) and IAC Rv (5′CTCCCGCAACAAACCCTATAAAT 3′) primers and 50 nM IAC Tq probe (5′ VIC-AGCATCTGTTCTTGAAGGT-NFQ-MGB 3′). Cycling conditions were a first step at 95˚C for 10 min followed by 40 cycles at 95°C for 15 seconds and 58°C for 1 minute. The amplifications were carried out in a ViiA7 Real-Time PCR System (Applied Biosystems, USA). Standard curves for the absolute quantification were constructed by serial dilution of DNA, extracted from 1 x 10^6^ trypomastigotes of *T*. *cruzi* (Dm28c-luc and Tulahuén strain), ranging from 10^5^ to 0.5 parasite equivalents (par. eq). Normalization of the parasite load was performed by tissue mass, after the absolute quantification of *T*. *cruzi* by real time qPCR and results were expressed as parasite equivalents/tissue mass (g).

### Statistical analyses

Kruskal-Wallis (Dunn’s post-test) or Mann-Whitney tests were used for the statistical analyses. P *values* < 0.05 were considered statistically significant. Tests were performed using GraphPad Prism 5.

## Results

### *Trypanosoma cruzi*-*luc* inoculation through the oral route promotes acute infection in mice

**Fig 1:**
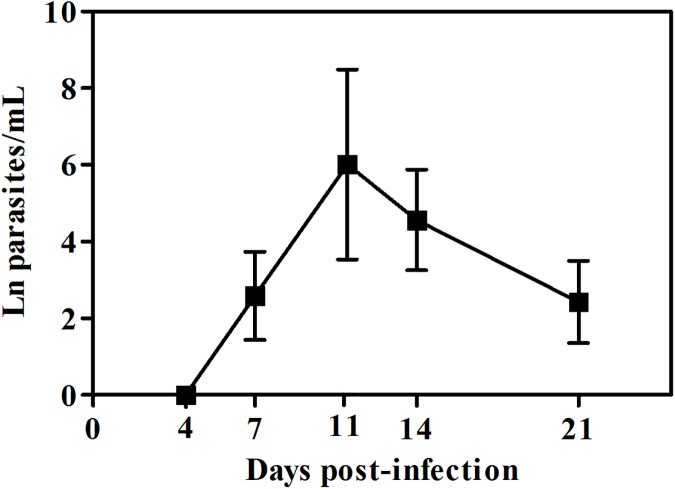
Parasitemia levels in mice orally infected with Dm28c-luc. Male BALB/c mice were infected with 1x10^6^ trypomastigotes forms of *T*. *cruzi* expressing luciferase (Dm28c-luc) through the oral cavity (**OI**). Parasitemia was assessed during acute phase by couting parasites with light microscope and the number of peripheral blood parasites was calculated by the Brener method. Values represent mean ± SEM. n: 4 and 11 dpi = 6; 7, 14 and 21 dpi = 16. The total number was obtained from two independent experiments.

Mice orally infected with *T*. *cruzi*Dm28c-luc were examined for blood parasitemia during the acute phase of infection. Peripheral blood parasites started to be detectable at 7 dpi, with a peak of parasitemia at 11 dpi. At later moments, the number of circulating parasites gradually decreased (Fig 1).

### The head region is the main affected area in orally *Trypanosoma cruzi* infected mice

**Fig 2:**
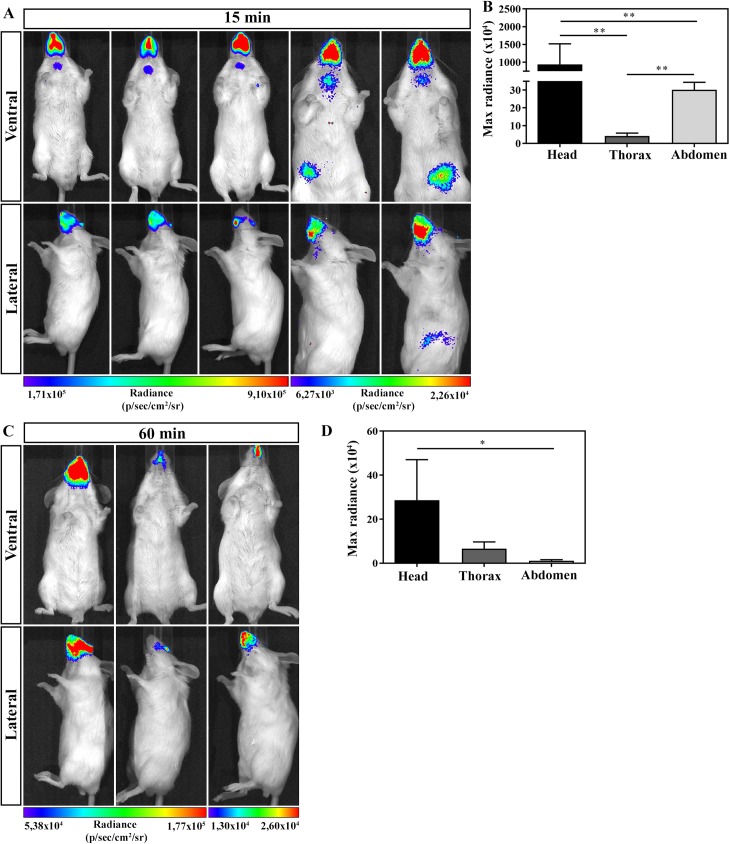
*In vivo* bioluminescence imaging of mice infected with Dm28c-luc at 15 and 60 min of infection. Male BALB/c mice were infected in the oral cavity (**OI**) with 1x10^6^ trypomastigotes forms of *T*. *cruzi* expressing luciferase (Dm28c-luc). Evaluation of **OI** mice was performed at 15 (A and B) and 60 min (C and D) post-infection using bioluminescent imaging (IVIS Lumina system). (A) *In vivo* bioluminescence imaging at 15 min post-infection (n = 5). (B) *In vivo* quantification of luminescent signal at 15 min post-infection (n = 5). (C) *In vivo* bioluminescence imaging at 60 min post-infection (n = 3). (D) *In vivo* quantification of luminescent signal at 60 min post-infection (n = 3). The scale bar for radiance (below) was correlated with the signal intensity, where red indicates higher signal and blue indicates a lower signal. Maximum and minimum signals are indicated at the right and left of the scale bar, respectively. Numbers represent mean ± SEM. Data were analyzed using one tailed Mann-Whitney test. Statistical analysis was performed using GraphPad Prism 5. * p<0.05; ** p < 0.01.

In order to determine the anatomical route of parasites entrance after **OI**, mice were infected and evaluated by bioluminescence imaging. At 15 and 60 min after **OI**, mice were placed inside the IVIS Lumina chamber and the images were obtained in ventral (upper panels) and lateral (lower panels) position (Fig 2). Detection of bioluminescence images after 15 min of **OI** showed that all infected mice analyzed had highest intensity of bioluminescence in the head region, concerning the mouth, nose and eyes. Although less intensive, bioluminescence was also observed in the neck, thorax and at the abdominal region. Bioluminescence signals were consistently observed from either ventral or lateral viewpoints (Fig 2A and 2B). One hour after infection, the major bioluminescence image detected remained in the head region (Fig 2C and 2D).

To confirm luciferase activity in living trypomastigotes, 5x10^4^ Dm28c-luc *T*. *cruzi* parasites were incubated *in vitro* with medium or D-luciferin in 24 well plate (black circle). Medium or D-luciferin (150 μg/mL) substrate was added to the well and, after 5 min of incubation, images were acquired. As demonstrated in S3 Fig, luminescent signals were only detected in D-luciferin treated parasites. Moreover, as *in vivo* controls, non-infected mice were treated with D-luciferin and bioluminescent signal analyzed. S4 and S5 Figs show that, in absence of *T*. *cruzi* infection, D-luciferin was incapable to promote bioluminescent signal.

### The nasal cavity is the site of parasite persistence in acute oral Chagas disease

**Fig 3:**
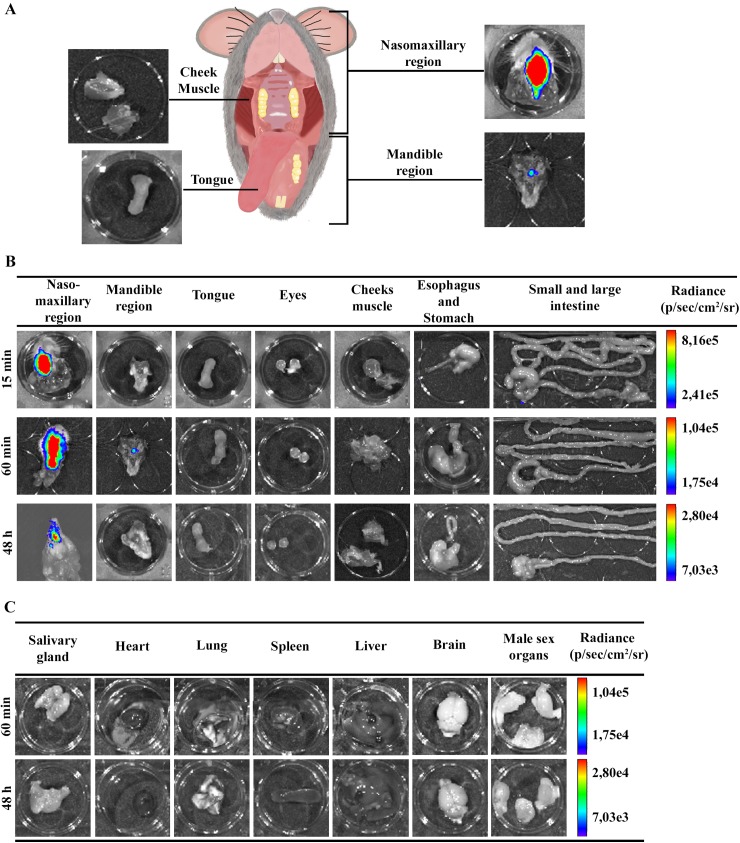
*Ex vivo* evaluation of dissected organs and tissues by bioluminescent imaging. Male BALB/c mice were infected in the oral cavity (**OI**) with 1x10^6^ trypomastigotes forms of *T*. *cruzi* expressing luciferase (Dm28c-luc). After 10 min of D-luciferin i.p administration (150 mg/kg), organs were harvested and images were captured using an IVIS Lumina II system. (A) Schematic picture for anatomic localization of organs and tissues analyzed. Nasomaxillary region includes all tissues from regions of the nose, nasal cavity and upper region of the oral cavity with exception of the cheek muscle. Mandible region includes all tissues of the mandible and the lower region of the oral cavity, with exception of the tongue (B and C). *Ex vivo* bioluminescence imaging from selected organs and tissues at 15 min (n = 3), 60 min (n = 4 in the nasomaxilary region; n = 2 in other organs) and 48 hours (n = 5) post-infection. The scale bar for radiance (right) was correlated with the signal intensity, where red indicates higher signal and blue indicates a lower signal. Maximum and minimum signals are indicated at the top and bottom of the scale bar, respectively.

For *ex vivo* evaluation of parasites in specific organs, mice were euthanized at 15 and 60 min and 48 hours after **OI**. The selected head tissues (nasomaxillary region, mandible region, cheek muscle, tongue and eyes) and gastrointestinal tract (esophagus, stomach, small and large intestine) were excised. The e*x vivo* evaluation of dissected organs and tissues by bioluminescence imaging confirmed the *in vivo* bioluminescent *T*. *cruzi* foci, as most of the signal detected was localized in the head, specifically in the nasomaxillary region (including areas of the nose, nasal cavity and upper oral cavity) (Fig 3A and 3B). A slight bioluminescence signal was observed in the cecum and mandible region in one single animal, 15 and 60 min after infection, respectively (Fig 3B). Furthermore, no luminescent signal was observed in tongue, eyes, cheek muscle, stomach and small intestine at this time (Fig 3B). At 60 min and 48 hours after **OI,** *ex vivo* bioluminescence imaging of the heart, brain, spleen, liver, male sex organs, lung and salivary gland was negative (Fig 3C). Taken together, our data suggests that the primary site of *T*. *cruzi* invasion due to **OI** is located at the upper region of the oral cavity, specifically at the nasomaxillary region. To exclude the possibility of an intranasal contamination in our oral infection protocol, mice were inoculated with black ink suspensions at the oral cavity or intranasally. As observed in S2 Fig orally inoculated mice after 5 min showed ink labeling in the tongue and the oral cavity, but were negative in the nasal cavity. In contrast, the intranasal inoculation clearly labeled the nasal cavity (S2 Fig).

**Fig 4:**
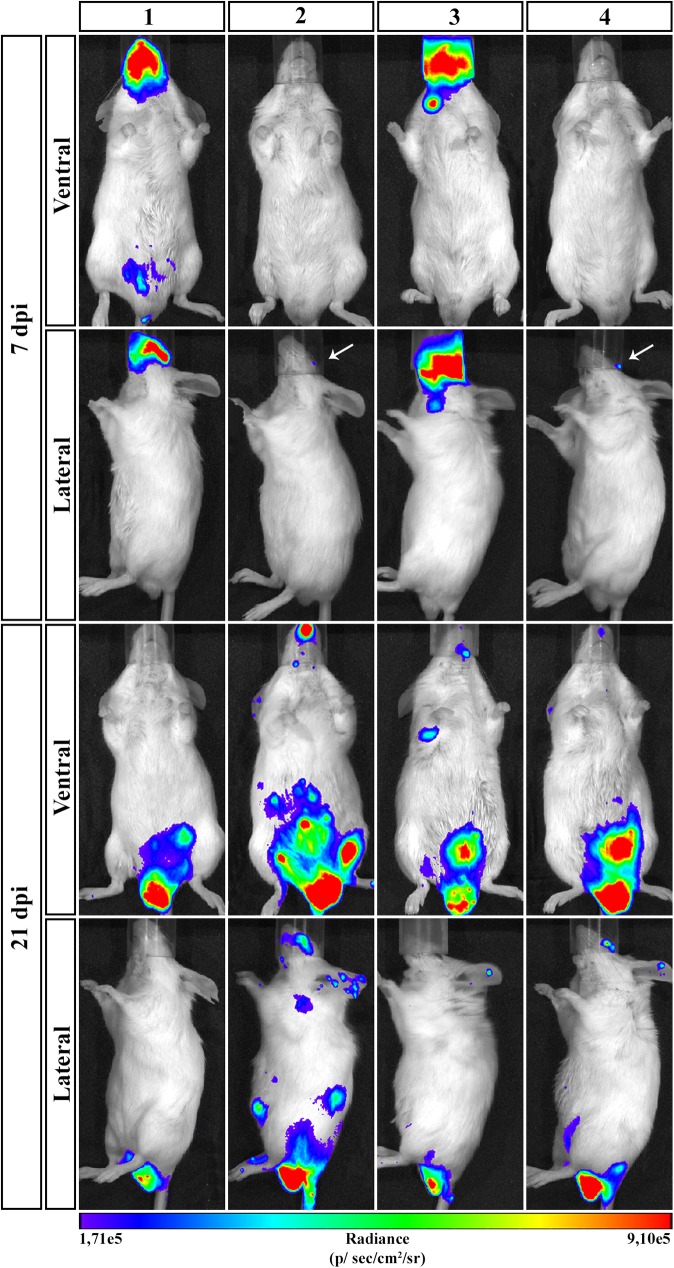
Course of parasite dissemination in *Trypanosoma cruzi* oral infection. Male BALB/c mice were infected in the oral cavity (**OI**) with 1x10^6^ trypomastigotes forms of *T*. *cruzi* expressing luciferase (Dm28c-luc). Representative *in vivo* bioluminescence images were acquired in the same mice (n = 6), at 7 and 21 dpi, after 15 min of D-luciferin **IP** administration (150 mg/kg), using IVIS Lumina image system (Xenogen) The scale bar for radiance (below) was correlated with the signal intensity, where red indicates higher signal and blue indicates a lower signal. Maximum and minimum signals are indicated at the right and left of the scale bar, respectively. White arrows indicate the presence of bioluminescence.

To have an overview of parasite distribution at different stages of infection, **OI** mice were analyzed at 7 dpi, an early stage of infection when blood parasites started to be detected, and at 21 dpi, a late point of the acute phase allowing a better analysis of parasite distribution and the target tissues. On 7 dpi, bioluminescent signal was detected in the head, neck and abdomen. It is noteworthy that the head region (mouth, nose and eyes) remained the major focus of bioluminescence (Fig 4). At 21 dpi, infection was dispersed trough the animal body, including head, ears, abdomen, genital region and thorax. Interestingly, at this moment, the genital region showed to be an important focus of bioluminescence signal (Fig 4).

To accurately identify the infected tissue, images of individual organs were captured at 7 and 21 dpi. Dissected tissues comprise the nasomaxillary region, palate, mandible, tongue, eyes, cheeks muscle, esophagus, stomach, small and large intestines, mandibular lymph nodes, salivary gland, heart, lung, spleen, liver, brain, pituitary gland, mesenteric fat and lymph nodes and male sex organ, including preputial glands, testicles, epididymis fat and penis. To better evaluate the nasomaxillary region, we removed the hard and soft palate exposing nasal septum and nasal cavity.

**Fig 5:**
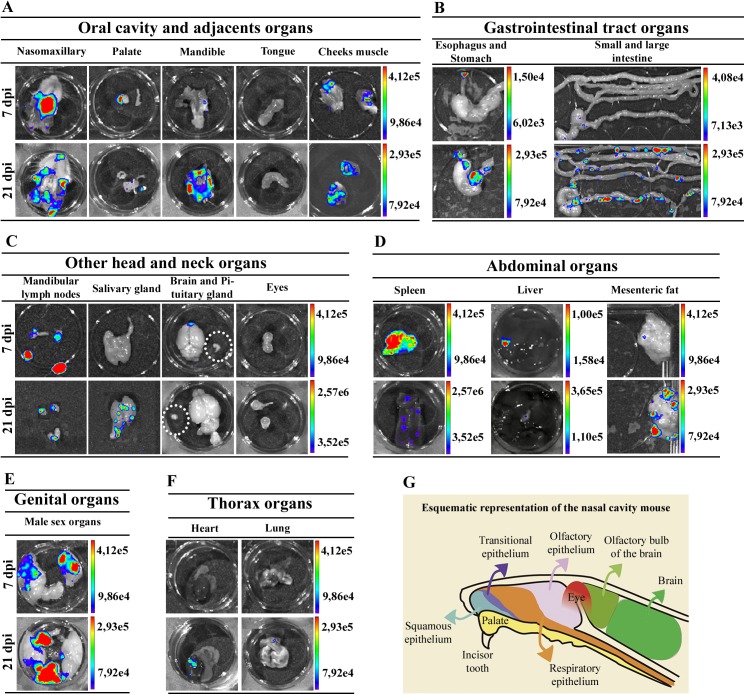
*Ex vivo* bioluminescence images from infected mice confirm the systemic dissemination of the parasite. Male BALB/c mice were infected in the oral cavity (**OI**) with 1x10^6^ trypomastigotes forms of *T*. *cruzi* expressing luciferase (Dm28c-luc). After 10 min of D-luciferin administration **IP** (150 mg/kg), organs were harvested and images were captured using an IVIS Lumina II system. *Ex vivo* bioluminescence imaging at 7 and 21 dpi: (A) oral cavity and adjacent organs; (B) gastrointestinal tract organs; (C) others head and neck organs. Pituitary gland: inside white circle; (D) abdominal organs; (E) In the male sex organ image, testicle and epididymal fat are located at the sides and the preputial gland in the bottom; (F) thorax organs. (n = 4 palate, cheek muscle; pituitary gland, mandibular lymph nodes mesenteric fat and lymph nodes; n = 6, others organs). The scale bar for radiance (right) was correlated with the signal intensity, where red indicates higher signal and blue indicates a lower signal. Maximum and minimum signals are indicated at the top and lower region of scale bar, respectively. (G) Schematic drawing of an anatomic section from a mouse head. The septum was removed, exposing the lateral wall and some of the major structures in the head. Palate (yellow), eyes (red), brain (light and dark green), olfactory bulb (light green) and nasal cavity are shown and the distribution of surface epithelial types lining the nasal airways is represented in blue, dark-purple, orange and light-purple colors for the squamous, transitional, respiratory, and olfactory epithelium, respectively.

**Fig 6:**
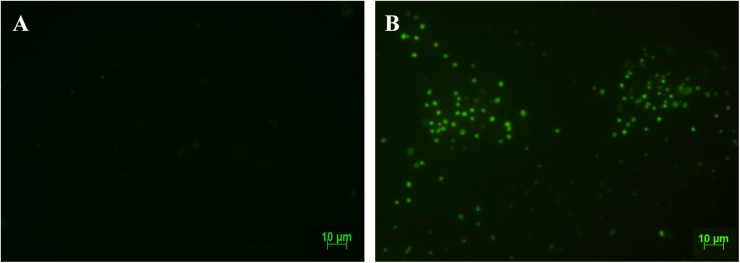
Amastigote nests detection by fluorescence microscopy of nasal cavity from mice infected with Dm28c-GFP. Male BALB/c mice were infected with 1x10^6^ trypomastigotes forms of *T*. *cruzi* expressing GFP reporter gene (Dm28c-GFP). At 6 dpi, the nasal cavity tissues were removed, frozen and sections were prepared for fluorescence microscopy analysis. (A) Representative fluorescence image of the nasal cavity from uninfected mice. (B) Representative fluorescence image of the nasal cavity from infected mice. Several amastigote nests (green) can be observed in the nasal cavity. Data represent analysis from an experiment with n = 2.

E*x vivo* evaluation of dissected organs and tissues at 7 dpi demonstrated that high bioluminescent signal remained at the nasomaxillary region of the mice (Fig 5A and S6 Fig). Furthermore, after removal of the entire palate, nasal cavity and nasal septum region showed the major bioluminescence signal (Fig 5A). Light foci were also detected in the palate in 75% of **OI** mice, shown in Table 1, which describes the percentage *T*. *cruzi*-positive tissues analyzed (Fig 5A and Table 1 and S6 Fig). Interestingly, at this moment of infection, images of *T*. *cruzi* were detected in the brain, located in the olfactory bulb region (Fig 5C and S6 Fig). Bioluminescence was also detected in the cheek muscle, mandibular lymph nodes and mandible in 50% of **OI** mice (Fig 5A and 5C and Table 1 and S6 Fig) and 66.6% of spleens (Fig 5D and Table 1). A slight bioluminescence signal was observed in the esophagus, liver, large and small intestines, mesenteric fat and lymph nodes (Fig 5B and 5D and S6 Fig). Bioluminescent foci were also detected in male sex organs, specifically in the testicle and epididymis fat in 33.33% of **OI** mice (Fig 5E and Table 1 and S6 Fig). The bioluminescence signal was undetected at this time in the tongue, eyes, stomach, pituitary gland, salivary gland, lung and heart (Fig 5A, 5B, 5C and 5F and S6 Fig). In agreement with initial bioluminescent images, a large number of *T*. *cruzi* Dm28c-GFP amastigote nests are detected in the nasal cavity of **OI** mice at 6 dpi (Fig 6).

**Table 1:** Percentage of *T*. *cruzi—*positive tissues analyzed by bioluminescence and qPCR methods.

		Times after infection					
		60 min		7 dpi		21 dpi	
Region	Tissues	BLI (n)	qPCR (n)	BLI (n)	qPCR (n)	BLI (n)	qPCR (n)
**Oral cavity and adjacent organs**	Nasal cavity	100% (4)	80% (5)	100% (6)	100% (5)	50% (4)	100% (4)
	Palate	NA	I	75% (4)	I	25% (4)	I
	Mandible	50% (2)	NA	50% (6)	NA	75%(4)	NA
	Cheeks muscle	ND (2)	NA	50% (4)	NA	100% (4)	NA
	Tongue	ND (2)	I	ND (6)	I	ND (4)	I
**Head and neck organs**	Mandibular LN	NA	75% (4)	50% (4)	100% (3)	25% (4)	100% (3)
	Salivary gland	ND (2)	NA	ND (6)	NA	75%(4)	NA
	Brain	ND (2)	ND (5)	66,6% (6)	20% (5)	ND (4)	100% (4)
	Pituitary gland	NA	ND (5)	ND (4)	80% (5)	ND (4)	100% (4)
	Eyes	ND (2)	NA	ND (6)	NA	ND (4)	NA
**Abdominal organs**	Spleen	ND (2)	ND (5)	66,6%(6)	100% (4)	25% (4)	100% (4)
	Liver	ND(2)	ND (5)	33,3%(6)	60% (5)	25% (4)	100% (4)
	Mesenteric fat and LN	NA	NA	25% (4)	NA	100%(4)	NA
**Gastrointestinal tract organs**	Esophagus	ND (2)	25% (4)	16,6% (6)	NA	50% (4)	100% (3)
	Stomach	ND (2)	75% (4)	ND (6)	25% (4)	50% (4)	100% (3)
	Small intestine	ND (2)	20% (5)	50% (6)	100% (3)	75% (4)	100% (4)
	Large intestine	ND (2)	20% (5)	16,6% (6)	ND (4)	75% (4)	100% (4)
**Genital organs**	Male sex organs	ND (2)	NA	33,3% (6)	NA	100% (4)	NA
**Thorax organs**	Heart	ND (2)	20% (5)	ND (6)	80% (5)	50% (4)	100% (4)
	Lung	ND (2)	NA	ND (6)	NA	25% (4)	NA

BLI: Bioluminescence imaging; n: number of animals; LN: Lymph nodes; NA- not analyzed; ND- not detected; I- PCR inhibition. The percentage of infected tissues was obtained from the number of tissues presenting bioluminescence signal or *T*. *cruzi* SatDNA amplification, over the total number of tissue (from different mice) analyzed.

At 21 dpi, bioluminescence was clearly observed in the nasomaxillary region, palate, mandible region, cheek muscle, esophagus, mandibular lymph nodes, spleen, liver, mesenteric fat and lymph nodes and male sex organ (Fig 5A, 5B, 5C, 5D and 5E and S7 Fig). The major affected tissues and organs in the genital region were penis and preputial gland (Fig 5E). In addition, tissues such as the salivary glands, heart and lung started to reveal parasite presence at this moment (Fig 5C and 5F and S7 Fig). At this time of infection, we also observed an increased signal of bioluminescence in the gastrointestinal tract, mostly in the stomach, intestines and mesenteric fat (Fig 5B and 5D and S7 Fig). Bioluminescence signal was observed in 75% of the intestines analyzed and in 50% of stomach and esophagus (Fig 5B and Table 1 and S7 Fig). Finally, at 21 dpi, the *ex vivo* evaluation revealed that parasites were disseminated to different organs of the body.

In conclusion, at 7 and 21 dpi, *T*. *cruzi* spreads to other parts of the body, infecting other organs. The persistence of bioluminescence signal emitted from the nasomaxillary region suggested the existence of a general maintenance of parasite proliferation in this region.

### Quantitative PCR detected parasite loads correlated with *ex vivo* bioluminescence and confirmed the nasal cavity as the major site of parasite burden

In contrast to the classical techniques, bioluminescence imaging is able to identify small foci of infection in the whole animal, but, in some cases, bioluminescent signal can be under detection limits. Quantitative real-time PCR (qPCR) is an accurate technique to evaluate the presence of parasites in tissues. To examine the parasite burden in target tissues, we collected tissues from orally infected mice at 60 min, 7 and 21 dpi and performed qPCR to compute parasite load. Initially, tissues of the oral cavity, the gastrointestinal tract and adjacent regions, such as the nasal cavity, tongue, palate, mandibular lymph nodes, esophagus, stomach, large and small intestines were all analyzed by qPCR.

**Fig 7:**
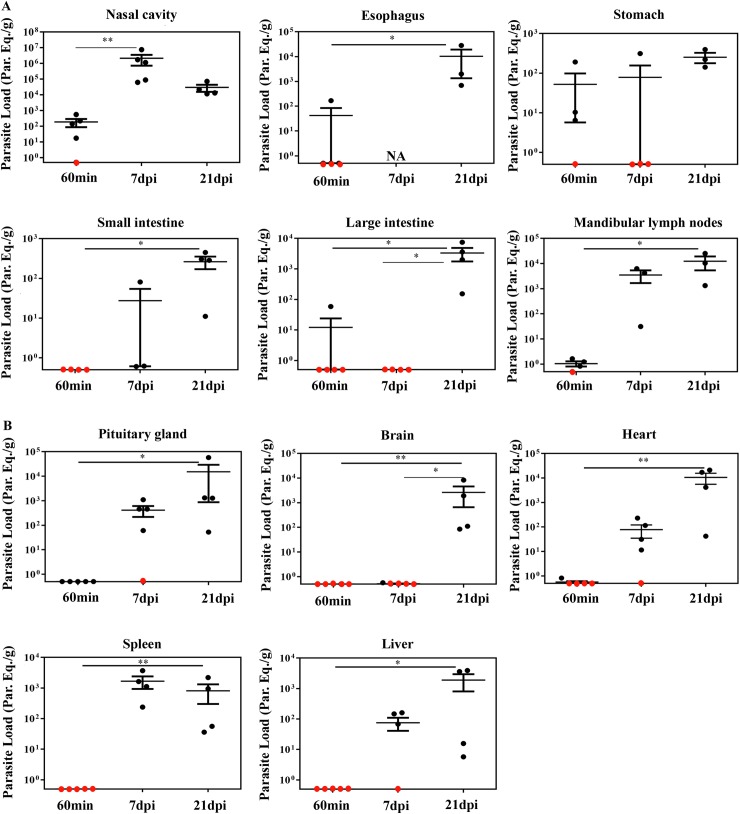
Quantification of tissue parasite loads in BALB/c mice orally infected with *T*. *cruzi* Dm28c-luc strain. Male BALB/c mice were infected in the oral cavity (**OI**) with 1x10^6^ trypomastigotes forms of *T*. *cruzi* expressing luciferase (Dm28c-luc). Organs and tissues were harvested for qPCR analysis to determine the parasite load (parasite equivalent/g) at 60 min, 7 and 21 dpi. The qPCR was performed in multiplex, targeting *T*. *cruzi* nuclear satellite DNA (Sat DNA) and IAC, as a quality control. (A) Parasite load in the nasal cavity (n: 60 min and 7dpi = 5; 21 dpi = 4), esophagus (n: 60 min = 4; 21 dpi = 3), stomach (n: 60 min and 7 dpi = 4; 21 dpi = 3), small intestine (n: 60 min = 5; 7 dpi = 3; 21 dpi = 4); large intestine (n: 60 min = 5; 7 and 21 dpi = 4) and mandibular lymph nodes (n: 60 min = 4; 7 and 21 dpi = 3). (B) Parasite load in the pituitary gland (n: 60 min and 7dpi = 5; 21 dpi = 4), brain (n: 60 min and 7 dpi = 5; 21 dpi = 4), heart (n: 60 min and 7dpi = 5; 21 dpi = 4), spleen (n: 60 min = 4; 7 and 21 dpi = 4) and liver (n: 60 min and 7 dpi = 5; 21 dpi = 4). Red dots: no parasite detection. Values present mean ± SEM. Kruskal-Wallis (Dunn’s post-test) was used for group kinetics. Statistical analysis was performed using Graph Pad Prism 5. * p < 0.05, **p < 0,01.

Consistent with the bioluminescence results observed in the nasomaxillary region at 60 min (Fig 3B) and 7 dpi (Fig 5A), *T*. *cruzi* foci were detected in elevated numbers at the nasal cavity by qPCR. The first hour after infection showed *T*. *cruzi* Sat DNA detection in the nasal cavity among 80% of **OI** mice, with parasite quantification up to 560 parasite equivalents/g (par.eq./g) (mean of 180) (Fig 7A and Table 1). Parasite amplification was also detected in the esophagus, stomach, small intestine and large intestine (Fig 7A), although these tissues were negative by bioluminescence imaging (Fig 3B). Interestingly, at 60 min, SatDNA detection was observed in one **OI** mouse at the esophagus, small intestine and large intestine (Fig 7A). Furthermore, *T*. *cruzi* SatDNA was detected in 75% of the analyzed **OI** mice in the stomach and mandibular lymph nodes at 60 min, with *T*. *cruzi* quantification up to 191.1 (mean of 52.0) and up to 1.63 (mean of 1.0) par.eqs./g, respectively (Fig 7A and Table 1).

In addition, SatDNA *T*. *cruzi* quantification in the nasal cavity was much higher at 7 dpi, ranging from 6.2x10^3^ to 7.5x10^6^ par.eqs./g (mean of 2.2x10^6^) (Fig 7A). In this time points after infection, nasal cavity showed the highest parasite load among the analyzed tissues. Interestingly, mandibular lymph nodes also showed high parasite loads, ranging from 31.2 to 6300 par.eqs./g (mean of 3.5 x 10^3^) (Fig 7A). It becomes evident that the mean parasite load detected in the nasal cavity was 10^3^ times higher than in the other organs (Fig 7A).

At 21 dpi, due to parasite dissemination, high levels of par.eqs./g were detected in all tissues (Fig 7A), in accordance to the bioluminescence imaging. In addition, it was not possible to detect parasite presence in the palate and tongue due to PCR inhibition (no amplification of the qualitative exogenous internal amplification control (IAC). To evaluate parasite dissemination throughout the body and to determine if there was any correlation with the bioluminescence signal, we analyzed parasite load in the pituitary gland, brain, heart, spleen and liver at 60 min, 7 and 21 dpi. *Ex vivo* imaging of the brain, spleen and liver did not reveal any bioluminescence signal at 60 min (Fig 3C). As expected, qPCR results confirmed the bioluminescence imaging and *T*. *cruzi* DNA amplification was undetectable in these organs (Fig 7B). Low amount of parasite detection was observed in the heart of a single animal (0.8 par.eq./g), at 60 min (Fig 7B). At 7 dpi, *T*. *cruzi* SatDNA was detected in the heart, spleen, liver and pituitary gland (Fig 7B). Finally, at 21 dpi, parasite dissemination favored *T*. *cruzi* detection in all analyzed tissues (Fig 7B).

*T*. *cruzi* is highly genetically diverse and currently six Discrete Typing Units (DTU), TcI to TcVI, are recognized [38]. TcI, TcII, TcIII, TcIV and TcVI genotype has been reported in oral transmission of acute Chagas disease [18–25]. Because of this biological polymorphism, different strains may present tropisms for distinct tissues (cardiac muscle, myoenteric plexuses in the esophagus and rectum and others tissues) and consequently differences in the clinical forms of the disease [40]. Due to this difference tissues tropism in *T*. *cruzi* strains, qPCR of gastrointestinal tract, nasal cavity and heart tissues from **OI** mice using a different strain (Tulahuén strain, DTU—TcVI) was performed to compute parasite load. Tissues were collected at 60 min and 7 dpi from **OI** mice.

**Fig 8:**
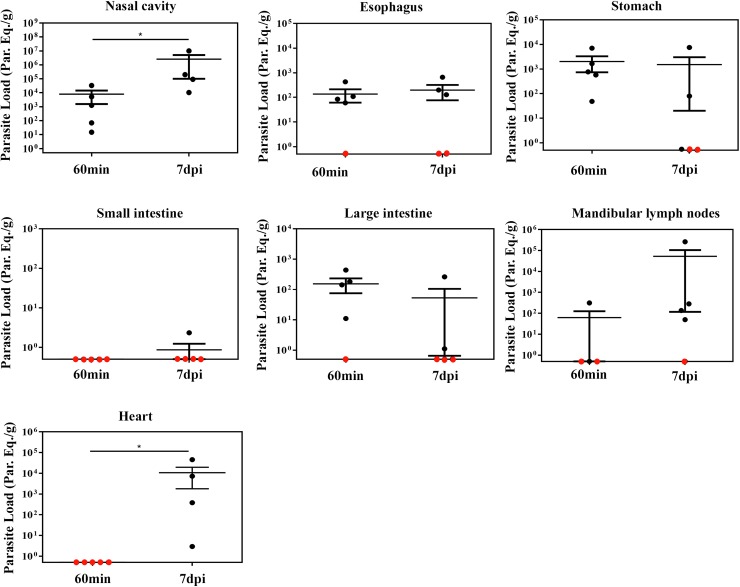
Quantification of tissue parasite loads in BALB/c mice orally infected with *T*. *cruzi* Tulahuén strain. Male BALB/c mice were infected in the oral cavity (**OI**) with 1x10^6^ trypomastigotes forms of *T*. *cruzi* Tulahuén strain (DTU-TcVI). Organs and tissues were harvested for qPCR analysis to determine the parasite load (parasite equivalent/g) at 60 min and 7 dpi. The qPCR was performed in multiplex, targeting *T*. *cruzi* nuclear satellite DNA (Sat DNA) and IAC, as a quality control. Parasite load in the: nasal cavity (n: 60 min = 5; 7 dpi = 4), esophagus (n: 60 min and 7dpi = 5), stomach (n: 60 min and 7 dpi = 5), small intestine (n: 60min and 7 dpi = 5), large intestine (n: 60 min and 7 dpi = 5), mandibular lymph nodes (n: 60 min = 4 and 7 dpi = 5) and heart (n: 60 min = 4 and 7 dpi = 5). Red dots: no parasite detection. Values present mean ± SEM. One tailed Mann-Whitney test was used for group kinetics. Statistical analysis was performed using GraphPad Prism 5. * p < 0.05.

Consistent with the qPCR results observed in **OI** mice with Dm28c-luc strain (DTU- TcI) (Fig 7), sixty minutes after infection, *T*. *cruzi* foci was detected in elevated numbers at the nasal cavity in **OI** mice with Tulahuén strain (DTU- TcVI). *T*. *cruzi* presence was also detected in the stomach at this time point (Fig 8). However, at 7dpi the highest SatDNA *T*. *cruzi* quantification in the nasal cavity suggested intense parasite growing in this tissue, in contrast with the stomach (Fig 8). Altogether, these data confirms that the nasal cavity is the preferential site *T*. *cruzi* infection and expansion in oral infection, regardless of DTU strain specificity (Fig 8).

Interestingly, the percentage of **OI** mice with blood parasitemia at 7 and 21 dpi was 25% and 56%, respectively. However, by assessing the percentage of infected mice in these same points of infection using bioluminescent imaging (evaluating the presence of the bioluminescence signal) and qPCR (evaluating *T*. *cruzi* SatDNA amplification in tissue), 100% of **OI** mice showed both bioluminescent signal and *T*. *cruzi* SatDNA amplification in tissues at 7 and 21 dpi. We conclude that the parasitemia is less sensitive to determine the percentage of infection in animals inoculated by the oral route in our model, since the bioluminescence techniques and qPCR showed signs of active infection in mice in these times.

**Fig 9:**
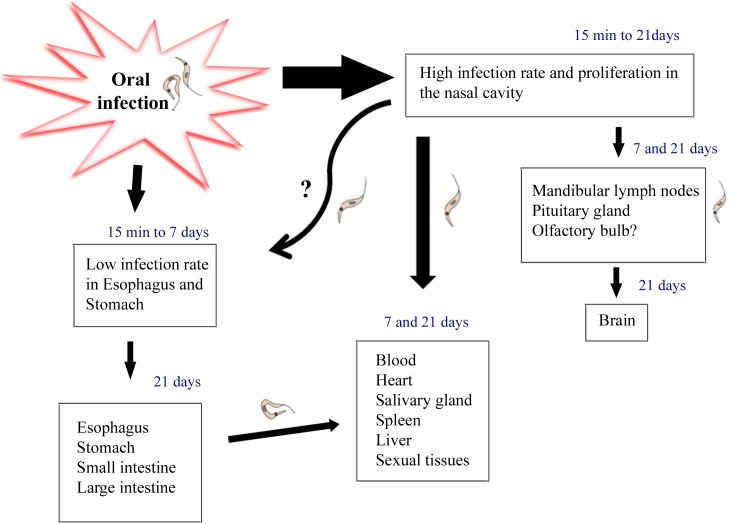
Hypothesis: Oral infection involves *Trypanosma cruzi* infection through the mouth into the nasal cavity, the main source of parasite replication. Nasal cavity parasites might disseminate through the olfactory nerve to the brain and also by the lymphatic and/or blood circulation to distant tissues. Moreover, as previously described by others, a small amount of parasites migrates to the gastric mucosa in initial moments of infection. *T*. *cruzi* infection in the stomach may also be associated to parasite dissemination trough the host.

Taken together, bioluminescence and qPCR data showed that at the first moments after **OI**, *T*. *cruzi* is able to infect nasal cavity, mandibular lymph nodes and stomach. However, nasal cavity is the major focus for parasite permanence and replication. These results show parasite distribution kinetics, thus suggesting that *T*. *cruzi* may disseminate to other organs (pituitary gland, brain, heart and liver) from the nasal cavity (Fig 9).

## Discussion

In the past years, the number of oral Chagas disease outbreaks in Brazil and other Latin America countries are increasing. Presently, the most common pathway of *T*. *cruzi* infection in the Brazilian Amazon is the oral route and, from 2000 to 2013, this pathway of infection was responsible for 70% of acute cases in Brazil [4, 6].These outbreaks were associated with ingestion of contaminated food and beverage[11, 41]. Orally infected patients are frequently highly symptomatic, presenting long-lasting fever, headache, facial and bipalpebral edema, lower limb edema, myalgia, abdominal pain, meningoencephalitis and the classical cardiac involvement [6, 9, 42–44]. Analysis of distinct outbreaks demonstrated that the mortality rate of affected patients in the first two weeks of infection is estimated at 8–35%, considerably higher than the mortality rate from the classical vectorial transmission (< 5–10%). The higher mortality rate can be associated with elevated prevalence of cardiac pathology and absence of an earlier diagnosis [14, 43].

Despite being an important route of infection, there are few studies regarding *T*. *cruzi* oral transmission in the literature. Previous data, using histopathology studies, showed signs of a possible *T*. *cruzi* penetration in the oral, esophageal, gastric, and intestinal mucosa with a local reaction with eosinophilia, infiltrated lymphocytes and monocytes after oral infection in dog [45]. In contrast, some authors have demonstrated that orally *T*. *cruzi* infected mice involves gastric mucosal invasion for the systemic infection. It has been shown, by histological analysis, that *T*. *cruzi* infection is observed in the gastric mucosal epithelium. However, parasites were not detected in other areas throughout the gastrointestinal tract, like esophagus and oropharynx. These authors observed that *T*. *cruzi* initiates systemic parasite dissemination as a consequence of an oral infection by gastric mucosal invasion [27]. By using intragastric or intrapharyngeal challenge, another group observed that *T*. *cruzi* glycoproteins, such as gp82 and gp30, are important for gastric invasion. Prior to invasion, the parasite binds to gastric mucin using these glycoproteins that allow *T*. *cruzi* to invade and replicate in the stomach [29, 31, 46, 47]. We have previously shown that the site of inoculation, through the oral cavity (**OI**) or the stomach (by gavage-**GI**), differentially affects host immune response and mortality. **OI** developed a highly severe acute disease with higher parasitemia, TNF serum levels, hepatitis and mortality rates when compared to **GI** [15], suggesting that the inoculum site is a key factor in Chagas disease progression, possibly modulating local immune mechanisms that impacts in the systemic immunity. In addition, intraperitoneal (**IP**), intravenous and subcutaneous infection shows higher infection rates and mortality than mucosal ones (oral, intragastric, intrarectal, genitalia or conjunctival) [33, 48, 49].

Here, we searched for the site of parasite entry in the host in orally infected mice. It is well accepted that bioluminescence imaging is an innovative technique that helps the identification of parasite distribution in distinct tissues, allowing a panoramic comprehension of *T*. *cruzi* dissemination in the entire animal body [34]. By using bioluminescence technique, we demonstrated that, few minutes after **OI**, parasites are concentrated in the head region, specifically in the nasomaxillary region (upper oral cavity, nose and nasal cavity). In a lesser intensity, parasites were also detected in the thorax and at the abdominal region. In addition, *T*. *cruzi* was detected in the nasal cavity and draining lymph nodes at 60 min post-infection by qPCR, confirming that the nasal cavity has the highest parasite load among affected tissues, in contrast with the stomach and intestines. In the same way, two and seven days after inoculums, images revealed that the nasomaxillary region remains as the major focus of infection.

Interestingly, facial edema is a common feature in affected patients, being described in 57–100% of cases in Brazilian outbreaks of oral infection [6]. Nevertheless, a contaminated sugar cane juice outbreak of oral infection in Paraiba State (Brazil) revealed the presence of bilateral palpebral edema in 92% of orally infected patients [44]. An outbreak with contaminated fresh guava juice in Venezuela showed that 40% of hospitalized patients had facial edema [50]. Moreover, another outbreak in Venezuela involving five members of the same family described that all patients showed edema in the face, mouth and cheek, and edema and paraesthesia of the tongue [51]. Furthermore, other clinical finding in the face region, such as gingivitis and dry cough has been attributed to the penetration of the parasite throughout the oral or pharyngeal cavity [6, 43]. Interestingly, *T*. *cruzi* infection and gingival inflammatory foci has been shown at the oral cavity from a chronic Chagas disease patient [52]. These findings might be associated to our present data, which describe for the first time the nasomaxillary region as the main target tissue following oral *T*. *cruzi* infection.

The mouth can be targeted by various infectious diseases, including viral, bacterial, and fungal. The oral cavity contains distinct mucosal surfaces composed of sophisticated structures and molecules, such as mucins, in which the microorganisms can bind and colonize the environmental cells [53]. It has been shown that the soft palate is an important site of infection and adaptation of influenza viruses. The soft palate infection may contribute to airborne transmission by providing a mucin-rich microenvironment and perhaps the initial region of infection. In fact, the expression of α 2,3 sialic acids and viral hemagglutinin ligand is detected on the soft palate in the regions of the oral surface, mainly at the basal cells, and the nasopharyngeal tissues from humans and ferret [54]. Interestingly, α 2,3 sialic acids are the main molecule involved in *T*. *cruzi* transialidase mediated binding. Transialidase has been considered as an important virulence factor of *T*. *cruzi*, due to its ability to reduce host cell immune response and mediate *T*. *cruzi* and host cells adhesion [55]. It has been shown that transialidase have adhesive capacity with host sialoglycans, generating “eat me” signals in epithelial cells, facilitating the parasite entry into non-phagocytic cells [56]. Based in these previous studies we can hypothesize that oral *T*. *cruzi* infection may occur on the palate, through the interaction of transialidase molecules in the parasite membrane with α 2,3 sialic acids residues present in the soft palate [54]. Other molecules may also be involved in *T*. *cruzi* adhesion with oral cavity cells, such as mucins and glycoproteins such as gp82, gp30, gp90 [57].

Seven days after infection reveals that nasal cavity, nasal septum region, palate, cheek muscles, mandible and mandibular lymph nodes are target tissues of the parasite. Surprisingly, the mean parasite load detected by qPCR in the nasal cavity of **OI** mice with Dm28c-luc (DTU- TcI), is 103 times higher than other tissues. This predominant *T*. *cruzi* detection in mouse nasal cavity is also observed in **OI** mice with other *T*. *cruzi* strain (Tulahuén strain, DTU- TcVI. Altogether this data suggesting that nasal cavity is the main site of *T*. *cruzi* maintenance and replication following oral infection.

In the line with our findings, Giddings and colleagues demonstrated that nasal cavity is the principal site of parasite infection and replication after conjunctival *T*. *cruzi* infection with Tulahuén strain (DTU-TcVI). The predominant invasion occurs through epithelia lining nasal cavity and nasolacrimal ducts. *T*. *cruzi* initially replicates within these sites and further spread to draining lymphoid organs with systemic dissemination. In the nasal cavity, parasites were detected in areas such as the submucosa of the epithelial lining the nasal septum, nasal mucosa-associated lymphoid tissue and bone marrow of the facial bones surrounding the nasal cavity [58]. Mice infected with the Tulahuén strain of *T*. *cruzi* by the intranasal route shows higher brain parasitism than mice infected by the subcutaneous pathway [49]. It was also observed that parasites gain access to the brain via olfactory nerve tissues. The authors proposed that, within the first moments, parasites invade nasal cavity cells, multiply and then migrate to the brain via the olfactory tissues [49]. Supporting this idea, we have observed that after infection and multiplication of parasites in the nasal cavity of orally infected mice, bioluminescence imaging of *T*. *cruzi* at 7 dpi were detected in the bulbous olfactory region of the brain in orally infected mice. Interestingly, parasites were also detected by qPCR in the pituitary gland at 7 and 21 dpi, but not in the central region of the brain at 7 dpi, turning positive at 21 dpi. Thus, we propose that brain infection is subsequent to the nasal cavity and the olfactory nerve tissue commitment. Corroborating our results of *T*. *cruzi* detection in the pituitary gland and in the brain, a previous study detected the parasite kinetoplast DNA in the pituitary gland during the acute phase [59].

Despite bioluminescence imaging is able to identify small foci of infection in the tissues and in the whole animal, this technique has limitations and some aspects that should be considered [34, 37]. The detection sensitivity is dependent on several factors, such as the level of luciferase expression, type of tissues, depth of labeled cells within the body and sensitivity of the detection system. Thus, in some cases, bioluminescent signal can be under the detection limit [37, 58–60]. As we have observed in our model, the percentages of *T*. *cruzi*-positive analyzed samples by bioluminescence and qPCR are different in some tissue (Table 1). Indeed in both pituitary gland and the heart at 7 dpi the presence of *T*. *cruzi* was not detected by bioluminescence, however it was detected by qPCR. This can be explained by higher sensibility of the qPCR compared to bioluminescence, as the qPCR allows detection of at least 0.5 equivalents parasites [61] and bioluminescence does not.

*T*. *cruzi* infection has been associated to disturbances in immune-endocrine systems, leading to activation in the hypothalamus–pituitary–adrenal (HPA) axis and high glucocorticoid production. The high glucocorticoid secretion seems to limit the excessive production of pro-inflammatory cytokines, protecting the host from tissue injury and metabolic alterations. Furthermore, the elevated glucocorticoid production in the acute phase is involved in thymus atrophy and immature T CD4^+^CD8^+^ cell apoptosis [60, 61].

In Fig 4 we observe that animals analyzed showed differences in bioluminescence signal. Some animals present less intensity of bioluminescence signal in the head, demonstrating that these animals have a lower parasitism in this region in that time point. Note that with 21dpi these same animals presented a larger signal in the region in the nasal cavity, which shows that they may have different evolution kinetics. This does not exclude the fact that they were infected and presented high intensity of signal at the same regions as the others, but not exactly at the same time. These differences between mice in *T*. *cruzi* infection can be observed also in parasitemia (Fig 1) or in parasitism load at different tissues (Figs 7 and 8). Interestingly, we can also see in Fig 7A a large difference in parasite load in the nasal cavity with 7 dpi between animals analyzed by qPCR, although not analyzed in the same animals bioluminescence.

Interestingly, with the development of the infection and spread of *T*. *cruzi*, we observed the presence of bioluminescence signal mainly in the male sexual organs (testicles, epedidimal fat, preputial gland and epididymis). As described in previous studies, male sex organs are frequently infected in *T*. *cruzi* experimental infections, including testes, penis, epididymis ducts and accessory sex glands (prostate, preputial gland and seminal vesicle) of mice infected by **IP** route [62–65]. In humans some cases of orchitis due to gonadal parasitism during the acute phase of Chagas disease have been described. Furthermore, clinical manifestations of sexual dysfunction such as decreased of libido, erection and ejaculation were reported [66–69]. Although the possibility of sexual transmission of *T*. *cruzi* has been suggested, few studies have been published on this theme. In the acute phase of experimental infection, sexual transmission has been described, but with low transmission rates in uninfected and immunosuppressed females through males infected by **IP** route [70]. Ribeiro and colleagues evaluated the potential of sexually transmission of *T*. *cruzi* in the chronic phase with infected males to uninfected females and *vice versa* by using mice infected via **IP** route. After copulation, 100% of the animals, both males as females seroconverted (ELISA and IF) and presented *T*. *cruzi* DNA in the heart and skeletal muscle [71].

In the present work, we have identified the site of *T*. *cruzi* initial invasion and replication after infection through the oral route. Our results demonstrated that oral infection involves *T*. *cruzi* passage through the mouth into the nasal cavity, where parasite replication occurs. Then, nasal cavity parasites might disseminate through the olfactory nerve tissues and blood to distant tissues (Fig 9). Thus, the proper oral cavity operates as a potential source of infection, and places the regional innate and adaptive immune systems as central players in the disease progression. Therefore, the elucidation of the tissue/organs targets and the molecular components regulating the establishment of oral *T*. *cruzi* infection is critical to understanding the pathogenesis of this current form of Chagas’ disease.

## Supporting information

S1 Fig Flowchart of the total number of mice used in each experiment. Male BALB/c mice, aged 6–8 weeks were used in all experiments and the number of animals used in each experiment performed on different time post-infection was demonstrated in the flowchart. n = number of animals, BLI = Bioluminescence imaging, LN = lymph nodes.
(TIF)

S2 Fig Black ink inoculation via oral or intranasal cavity. Oral and intranasal inoculations were performed using black ink suspension. Animals were analyzed after 5 min of inoculation, the nasomaxillary region; tongue and nasal cavity were removed. To evaluate the nasal cavity we removed the hard and soft palate exposing nasal septum and nasal cavity (n = 2).
(TIF)

S3 Fig Bioluminescence of Dm28c-luc *Trypanosoma cruzi* trypomastigotes. *In vitro* activity of luciferase of *Trypanosoma cruzi* Dm28c-luc strain. In a 24-well plate, 5x10^4^ trypomastigotes were plated with D-luciferin (black circle) and negative control with medium RPMI with 10% FBS (white circle). 150 μg / ml of D-luciferin substrate was added to the well and after 5 min of incubation, image was acquired by IVIS Lumina system (Xenogen Corp., CA, USA). The scale bar for radiance (below) was correlated with the signal intensity, where red indicates higher signal and blue indicates a lower signal. Maximum and minimum signals are indicated at the top at the right and left of the scale bar, respectively.
(TIF)

S4 Fig *Ex vivo* evaluation of dissected organs and tissues from non-infected mice by bioluminescence imaging. Organs and tissues were removed after 10 min of D-luciferin (150 mg/kg) **IP** administration from non-infected mice and images were acquired using IVIS Lumina II system.
(TIF)

S5 Fig *In vivo* bioluminescence imaging from non-infected mice. Male BALB/c mice were inoculated with D-luciferin substrate, after 15 min of D-luciferin (150 mg/kg) **IP** administration images were acquired using IVIS Lumina II system. No background was visualized. The scale bar for radiance (below) was correlated with the signal intensity, where red indicates higher signal and blue indicates a lower signal. Maximum and minimum signals are indicated at the top at the right and left of the scale bar, respectively.
(TIF)

S6 Fig *Ex vivo* tissues bioluminescence images from OI mice at 7 dpi. Male BALB/c mice were infected in the oral cavity (**OI**) with 1x10^6^ trypomastigotes forms of *T*. *cruzi* expressing luciferase (Dm28c-luc). After 10 min of D-luciferin **IP** administration (150 mg/kg), organs were harvested and images were captured using an IVIS Lumina II system. *Ex vivo* tissues bioluminescence imaging at 7 dpi of nasomaxillary region (n = 6), palate (n = 4), mandible (n = 6), tongue (n = 6), cheek muscle (n = 4), esophagus and stomach (n = 6), small intestine and large intestine (n = 6) male sex organs (n = 6), mandibular lymph nodes (n = 4), salivary gland (n = 6), brain (n = 6) and pituitary gland (n = 4), eyes (n = 6), spleen (n = 6), liver (n = 6), mesenteric fat and lymph nodes (n = 4), heart (n = 6) and lung (n = 6). In the male sex organ image, testicle and epididymal fat are located at the sides and the preputial gland in the bottom. Pituitary gland: inside white circle. The scale bar for radiance (right) was correlated with the signal intensity, where red indicates higher signal and blue indicates a lower signal. Maximum and minimum signals are indicated at the top and lower of scale bar, respectively. White arrows indicate the presence of bioluminescence.
(TIF)

S7 Fig *Ex vivo* tissues bioluminescence images from OI mice at 21 dpi. Male BALB/c mice were infected in the oral cavity (**OI**) with 1x10^6^ trypomastigotes forms of *T*. *cruzi* expressing luciferase (Dm28c-luc). After 10 min of D-luciferin **IP** administration (150 mg/kg), organs were harvested and images were captured using an IVIS Lumina II system. *Ex vivo* bioluminescence imaging at 21 dpi of nasomaxillary region (n = 6), palate (n = 4), mandible (n = 6), tongue (n = 6), cheek muscle (n = 4), esophagus and stomach (n = 6), small intestine and large intestine (n = 6) male sex organs (n = 6), mandibular lymph nodes (n = 4), salivary gland (n = 6), brain (n = 6) and pituitary gland (n = 4), eyes (n = 6), spleen (n = 6), liver (n = 6), mesenteric fat and lymph nodes (n = 4), heart (n = 6) and lung (n = 6). In the male sex organ image, testicle and epididymal fat are located at the sides and the preputial gland in the bottom. Pituitary gland: inside white circle. The scale bar for radiance (right) was correlated with the signal intensity, where red indicates higher signal and blue indicates a lower signal. Maximum and minimum signals are indicated at the top and lower scale bar, respectively. White arrows indicate the presence of bioluminescence.
(TIF)
