# Supplementary figures and images for: Unraveling Chagas disease transmission through the oral route: Gateways to Trypanosoma cruzi infection and target tissues
Source: PLoS Negl Trop Dis. 2017 Apr 5;11(4):e0005507. doi: 10.1371/journal.pntd.0005507 (PMC5397068; doi:10.1371/journal.pntd.0005507)

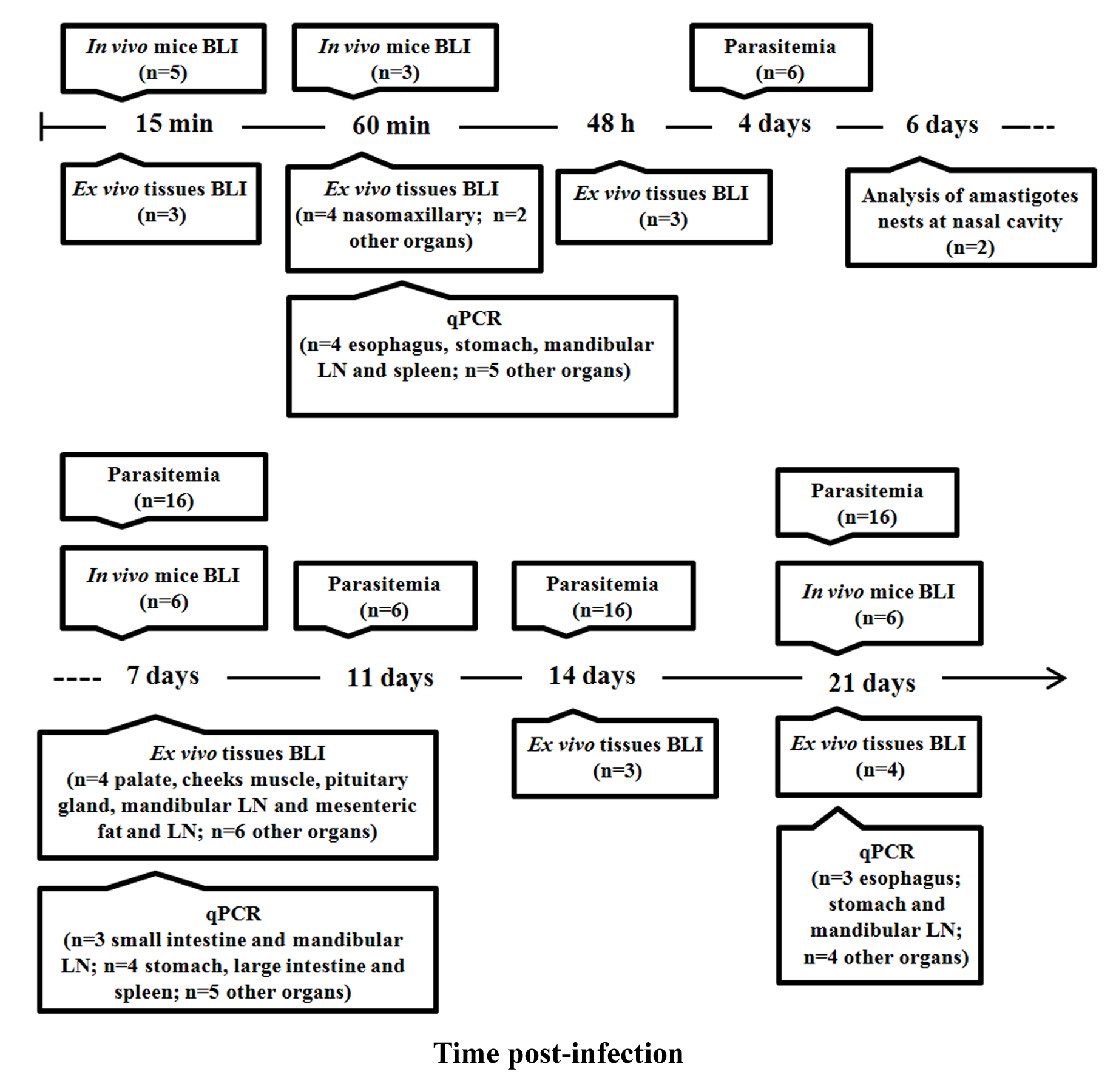

Supplement: S1 Fig — Male BALB/c mice, aged 6–8 weeks were used in all experiments and the number of animals used in each experiment performed on different time post-infection was demonstrated in the flowchart. n = number of animals, BLI = Bioluminescence imaging, LN = lymph nodes. (TIF) [file pntd.0005507.s001.tif]

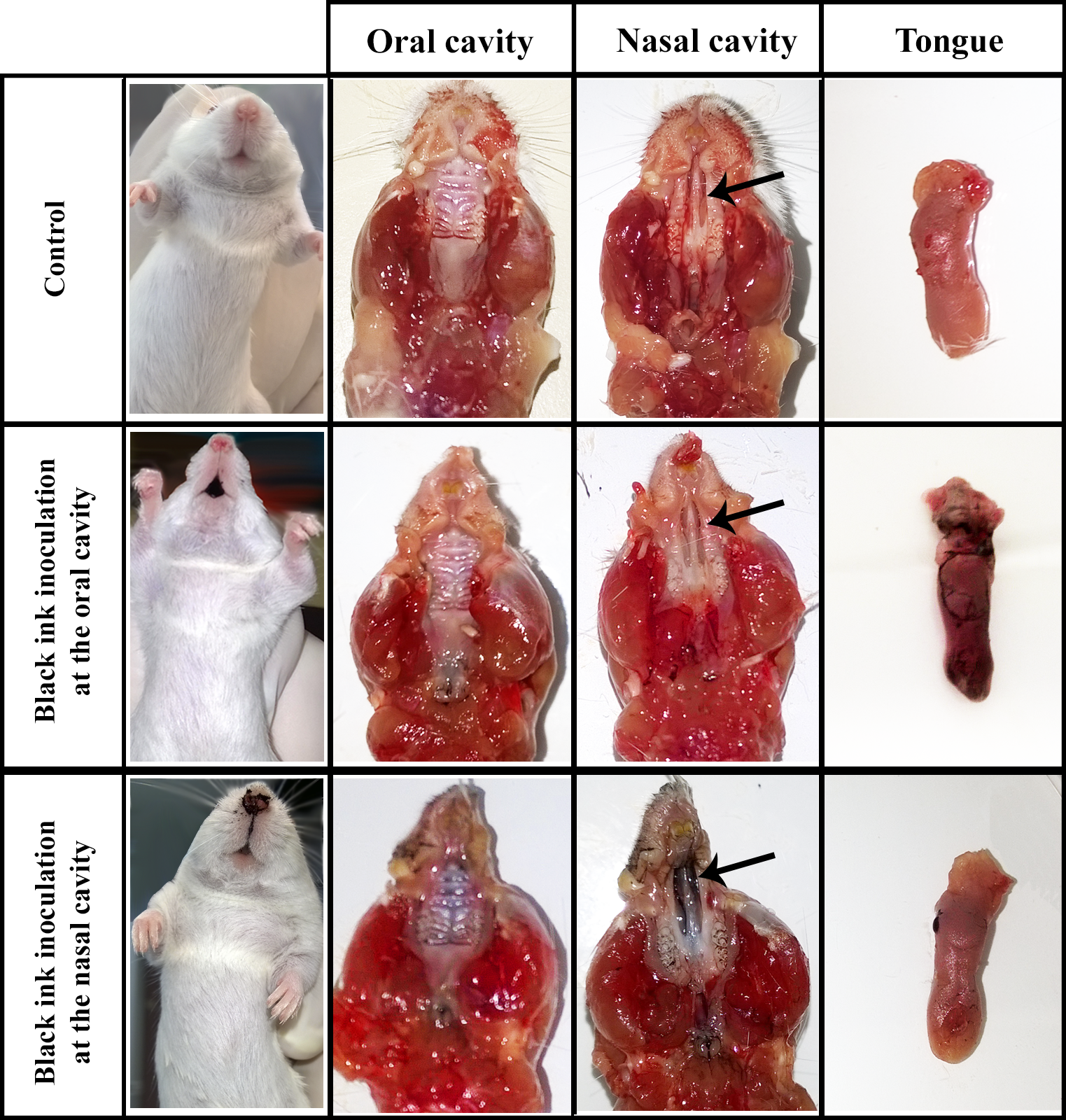

Supplement: S2 Fig — Oral and intranasal inoculations were performed using black ink suspension. Animals were analyzed after 5 min of inoculation, the nasomaxillary region; tongue and nasal cavity were removed. To evaluate the nasal cavity we removed the hard and soft palate exposing nasal septum and nasal cavity (n = 2). (TIF) [file pntd.0005507.s002.tif]

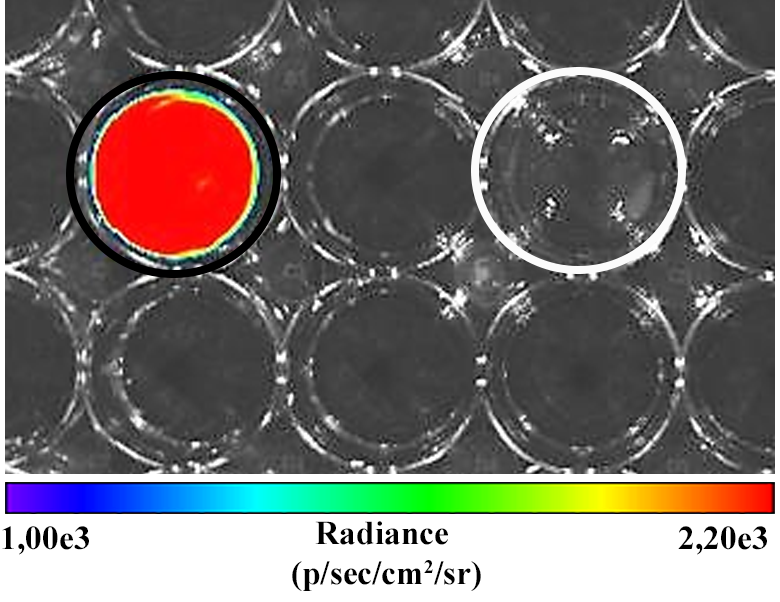

Supplement: S3 Fig — In vitro activity of luciferase of Trypanosoma cruzi Dm28c-luc strain. In a 24-well plate, 5x10^4^ trypomastigotes were plated with D-luciferin (black circle) and negative control with medium RPMI with 10% FBS (white circle). 150 μg / ml of D-luciferin substrate was added to the well and after 5 min of incubation, image was acquired by IVIS Lumina system (Xenogen Corp., CA, USA). The scale bar for radiance (below) was correlated with the signal intensity, where red indicates higher signal and blue indicates a lower signal. Maximum and minimum signals are indicated at the top at the right and left of the scale bar, respectively. (TIF) [file pntd.0005507.s003.tif]

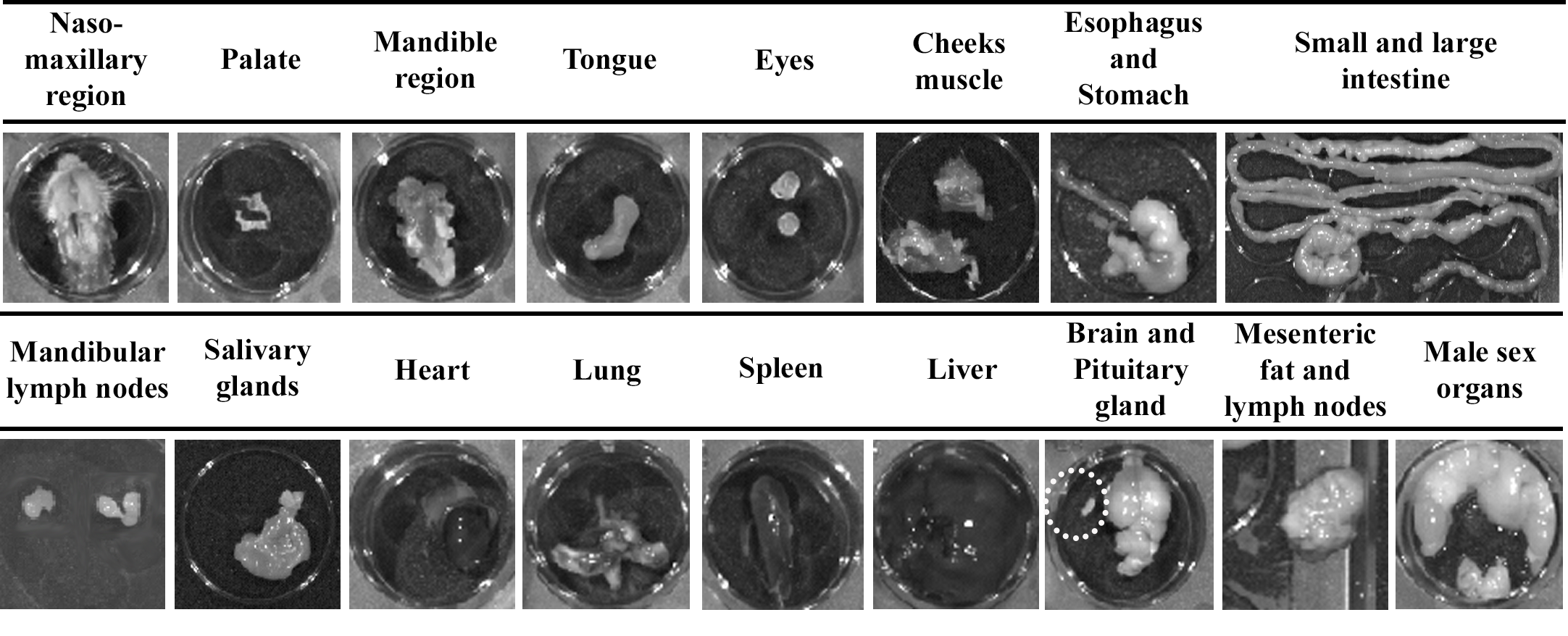

Supplement: S4 Fig — Organs and tissues were removed after 10 min of D-luciferin (150 mg/kg) IP administration from non-infected mice and images were acquired using IVIS Lumina II system. (TIF) [file pntd.0005507.s004.tif]

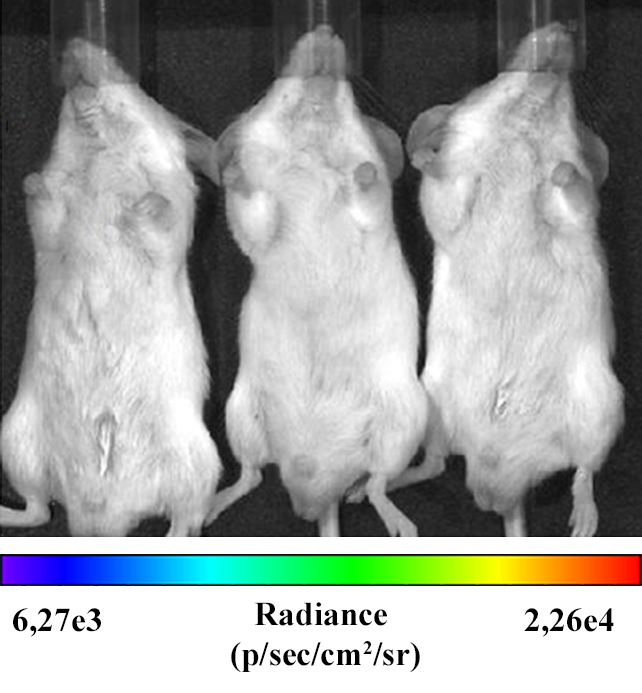

Supplement: S5 Fig — Male BALB/c mice were inoculated with D-luciferin substrate, after 15 min of D-luciferin (150 mg/kg) IP administration images were acquired using IVIS Lumina II system. No background was visualized. The scale bar for radiance (below) was correlated with the signal intensity, where red indicates higher signal and blue indicates a lower signal. Maximum and minimum signals are indicated at the top at the right and left of the scale bar, respectively. (TIF) [file pntd.0005507.s005.tif]

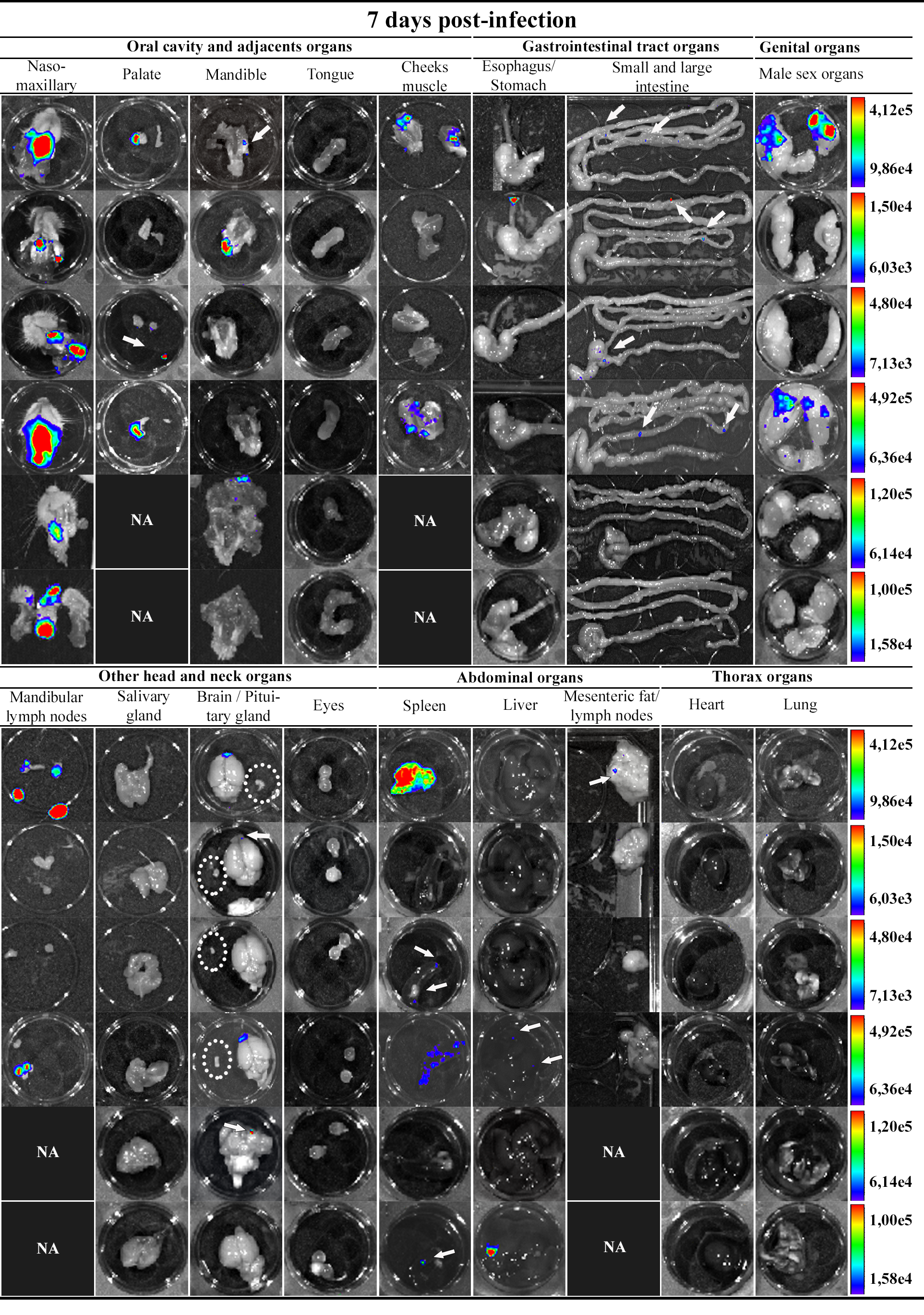

Supplement: S6 Fig — Male BALB/c mice were infected in the oral cavity (OI) with 1x10^6^ trypomastigotes forms of T. cruzi expressing luciferase (Dm28c-luc). After 10 min of D-luciferin IP administration (150 mg/kg), organs were harvested and images were captured using an IVIS Lumina II system. Ex vivo tissues bioluminescence imaging at 7 dpi of nasomaxillary region (n = 6), palate (n = 4), mandible (n = 6), tongue (n = 6), cheek muscle (n = 4), esophagus and stomach (n = 6), small intestine and large intestine (n = 6) male sex organs (n = 6), mandibular lymph nodes (n = 4), salivary gland (n = 6), brain (n = 6) and pituitary gland (n = 4), eyes (n = 6), spleen (n = 6), liver (n = 6), mesenteric fat and lymph nodes (n = 4), heart (n = 6) and lung (n = 6). In the male sex organ image, testicle and epididymal fat are located at the sides and the preputial gland in the bottom. Pituitary gland: inside white circle. The scale bar for radiance (right) was correlated with the signal intensity, where red indicates higher signal and blue indicates a lower signal. Maximum and minimum signals are indicated at the top and lower of scale bar, respectively. White arrows indicate the presence of bioluminescence. (TIF) [file pntd.0005507.s006.tif]

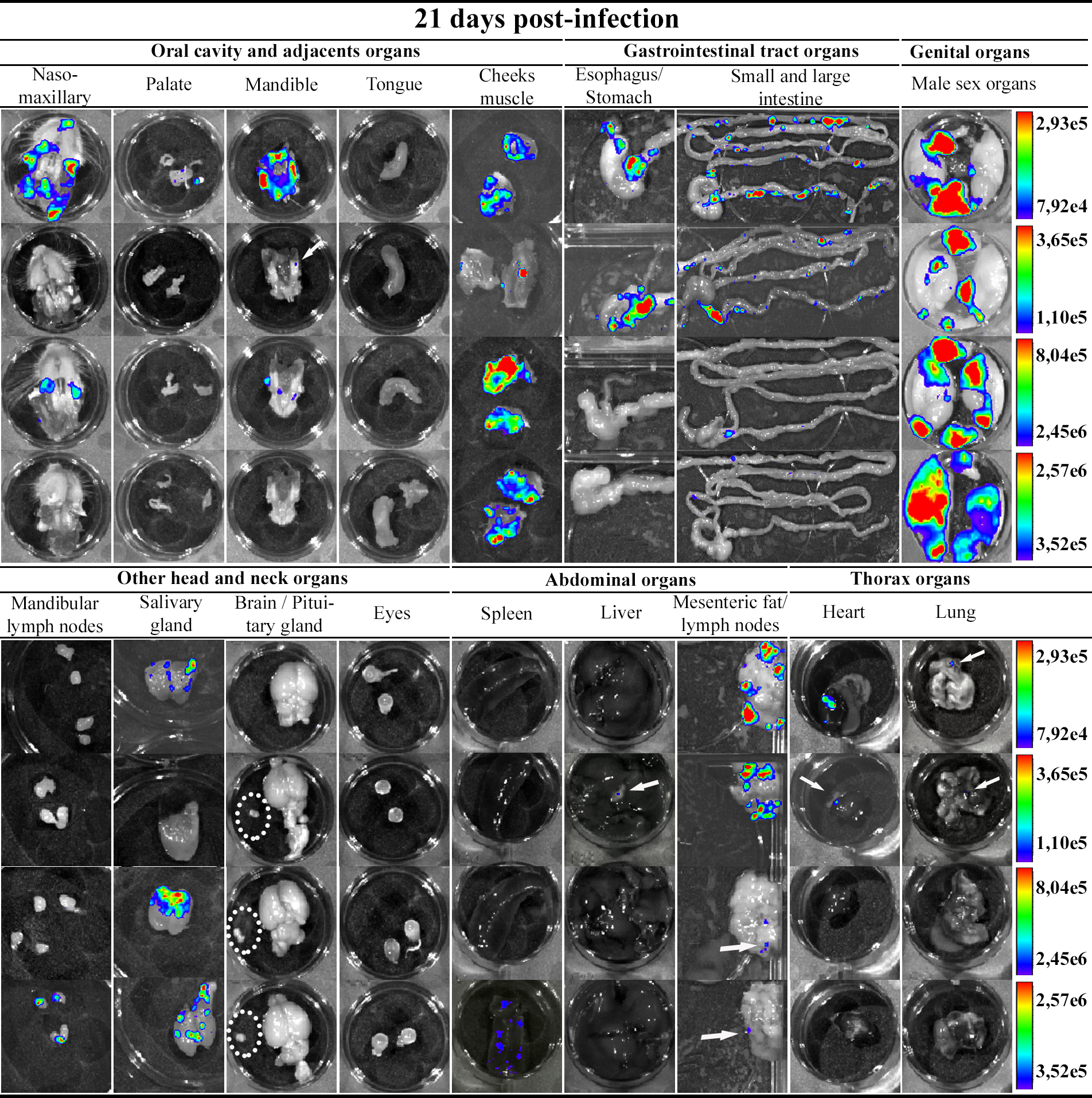

Supplement: S7 Fig — Male BALB/c mice were infected in the oral cavity (OI) with 1x10^6^ trypomastigotes forms of T. cruzi expressing luciferase (Dm28c-luc). After 10 min of D-luciferin IP administration (150 mg/kg), organs were harvested and images were captured using an IVIS Lumina II system. Ex vivo bioluminescence imaging at 21 dpi of nasomaxillary region (n = 6), palate (n = 4), mandible (n = 6), tongue (n = 6), cheek muscle (n = 4), esophagus and stomach (n = 6), small intestine and large intestine (n = 6) male sex organs (n = 6), mandibular lymph nodes (n = 4), salivary gland (n = 6), brain (n = 6) and pituitary gland (n = 4), eyes (n = 6), spleen (n = 6), liver (n = 6), mesenteric fat and lymph nodes (n = 4), heart (n = 6) and lung (n = 6). In the male sex organ image, testicle and epididymal fat are located at the sides and the preputial gland in the bottom. Pituitary gland: inside white circle. The scale bar for radiance (right) was correlated with the signal intensity, where red indicates higher signal and blue indicates a lower signal. Maximum and minimum signals are indicated at the top and lower scale bar, respectively. White arrows indicate the presence of bioluminescence. (TIF) [file pntd.0005507.s007.tif]
